# Three-Dimensional Collagen I Promotes Gemcitabine Resistance In Vitro in Pancreatic Cancer Cells through HMGA2-Dependent Histone Acetyltransferase Expression

**DOI:** 10.1371/journal.pone.0064566

**Published:** 2013-05-16

**Authors:** Surabhi Dangi-Garimella, Vaibhav Sahai, Kazumi Ebine, Krishan Kumar, Hidayatullah G. Munshi

**Affiliations:** 1 Division of Hematology/Oncology, Department of Medicine, Feinberg School of Medicine, Northwestern University, Chicago, Illinois, United States of America; 2 Jesse Brown VA Medical Center, Northwestern University, Chicago, Illinois, United States of America; 3 The Robert H. Lurie Comprehensive Cancer Center of Northwestern University, Northwestern University, Chicago, Illinois, United States of America; Fox Chase Cancer Center, United States of America

## Abstract

Pancreatic ductal adenocarcinoma (PDAC) is associated with a pronounced collagen-rich stromal reaction that has been shown to contribute to chemo-resistance. We have previously shown that PDAC cells are resistant to gemcitabine chemotherapy in the collagen microenvironment because of increased expression of the chromatin remodeling protein high mobility group A2 (HMGA2). We have now found that human PDAC tumors display higher levels of histone H3K9 and H3K27 acetylation in fibrotic regions. We show that relative to cells grown on tissue culture plastic, PDAC cells grown in three-dimensional collagen gels demonstrate increased histone H3K9 and H3K27 acetylation, along with increased expression of p300, PCAF and GCN5 histone acetyltransferases (HATs). Knocking down HMGA2 attenuates the effect of collagen on histone H3K9 and H3K27 acetylation and on collagen-induced p300, PCAF and GCN5 expression. We also show that human PDAC tumors with HMGA2 demonstrate increased histone H3K9 and H3K27 acetylation. Additionally, we show that cells in three-dimensional collagen gels demonstrate increased protection against gemcitabine. Significantly, down-regulation of HMGA2 or p300, PCAF and GCN5 HATs sensitizes the cells to gemcitabine in three-dimensional collagen. Overall, our results increase our understanding of how the collagen microenvironment contributes to chemo-resistance in vitro and identify HATs as potential therapeutic targets against this deadly cancer.

## Introduction

Despite tremendous efforts, the progress made in the treatment of pancreatic ductal adenocarcinoma (PDAC) has been frustratingly scant [1], [2]. PDAC continues to remain the fourth leading cause of cancer-related deaths in the US, with an ∼80% one-year mortality for most patients [3]. This lack of progress is in part due to the pronounced collagen-rich fibrotic reaction associated with PDAC tumors [4], [5], which subsequently limits the delivery and efficacy of chemotherapy [6], [7], [8], [9]. Recently, we published that PDAC cells in the three-dimensional collagen microenvironment induce high mobility group A2 (HMGA2), an architectural protein that regulates chromatin structure and also mediates chemo-resistance in the collagen-rich microenvironment [6], [10], [11]. Significantly, HMGA2 is upregulated in human PDAC tumors, particularly in high-grade tumors with lymph node metastases [12], [13].

PDAC is also associated with epigenetic changes, which have been linked to patient prognosis [14], [15]. Post-translational histone modification patterns detected by immunohistochemistry were shown to be predictive of prognosis in two large cohorts of PDAC patients treated with chemotherapy [14], [15]. PDAC patients whose tumors demonstrated a low expression of histone H3 lysine 27 tri-methylation (H3K27Me^3^) or histone H3 lysine 9 di-methylation (H3K9Me^2^), which are marks of closed chromatin (‘heterochromatin’) and gene repression [16], [17], [18], had significantly shorter overall survival than PDAC patients whose cancers displayed high histone H3K27Me^3^ or histone H3K9Me^2^ expression [14], [15]. However, PDAC patients with low histone H3K4Me^2^, which is a mark of a more open chromatin (‘euchromatin’) state, also demonstrated shorter overall survival than PDAC patients whose cancers displayed high H3K4Me^2^ expression [15]. In contrast to histone methylation, which is associated with both gene activation and repression, histone acetylation has only been linked with gene activation associated with the euchromatin state [16], [17], [18]. Despite the clear link between histone acetylation and cancer development [19], [20], the contribution of HATs to PDAC progression has not been well studied.

The expression and activity of HAT proteins are altered in a variety of cancers [21], [22]. For example, the p300 HAT is involved in activation of the c-myc promoter in PDAC cells [23]. The p300 HAT is also required for G1/S cell cycle transition, as downregulation of p300 HAT causes growth inhibition of melanoma cells [24]. HATs also modulate the chromatin state in cells, with GCN5 and PCAF HATs being usually required for global histone H3K9 acetylation and the p300 HAT being usually involved in global histone H3K27 acetylation [22], [25]. Interestingly, the GCN5 HAT contributes to widespread maintenance of active chromatin induced by the myc oncoprotein [26].

In this report, we examine the role and regulation of p300, PCAF and GCN5 HATs in PDAC cells. We show that the three-dimensional collagen microenvironment through HMGA2 expression promotes histone H3K9 and H3K27 acetylation along with p300, PCAF and GCN5 HAT expression in PDAC cells. Additionally, we show that human PDAC tumors with increased fibrosis display higher histone H3K9 and H3K27 acetylation, and have increased HMGA2 expression. Moreover, PDAC cells in three-dimensional collagen gels demonstrate increased protection against gemcitabine. Significantly, downregulating HMGA2 or p300, PCAF and GCN5 HATs sensitizes the cells to gemcitabine in three-dimensional collagen. Overall, our results increase our understanding of how the three-dimensional collagen microenvironment contributes to chemo-resistance in vitro, and establish HATs as potential therapeutic targets against this deadly cancer.

## Results

### Collagen increases histone H3K9 and H3K27 acetylation

**Figure 1:**
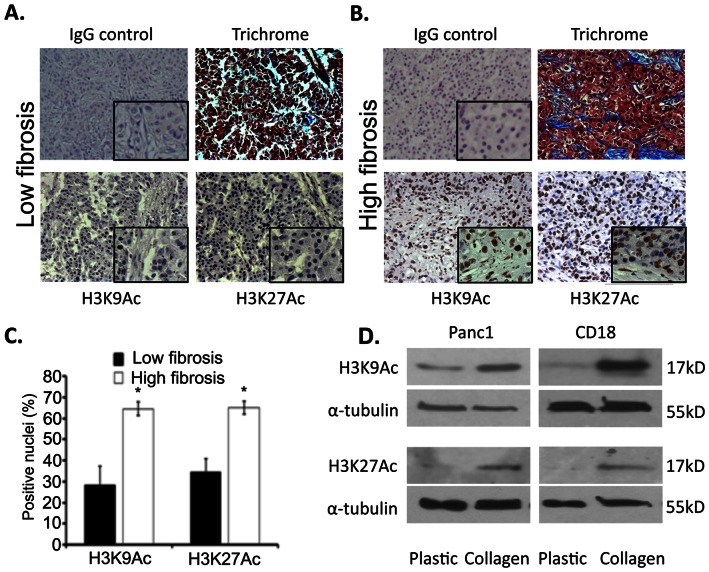
Collagen increases histone H3K9 and H3K27 acetylation. *A, B*. Human pancreatic tissue microarrays (TMAs) containing 24 specimens were immunostained with IgG control antibody or for histone H3K9 and histone H3K27 acetylation (Ac). The TMAs were also trichrome stained to assess for fibrosis. The *insets* show higher magnification images of staining with control IgG, and for histone H3K9Ac and histone H3K27Ac. *C*. Quantification of histone H3K9Ac- and histone H3K27Ac-positive cells was performed using Adobe Photoshop CS3 software. *, p<0.01 relative to sections with low fibrosis. *D*. Panc1 and CD18 cells were grown on tissue culture plastic or in three-dimensional collagen gels for 24 hours. Cells were lysed and immunoblotted for histone H3K9Ac and H3K27Ac using α-tubulin as loading control. The results are representative of at least four independent experiments.

Recently we published that PDAC cells growing in the collagen-rich microenvironment were protected against the effects of chemotherapy [6]. We showed that the chemo-protection was due to increased expression of HMGA2 [6], an architectural protein involved in regulating the chromatin state [10]. Since HATs have been linked with changes in the chromatin state and also mediate the response to DNA damage [19], [20], [21], [22], [27], [28], we examined whether fibrosis in human PDAC tumors was associated with changes in histone acetylation. Moreover, since p300 and GCN5 HATs are involved in chromatin relaxation by promoting acetylation at sites of DNA damage and facilitating repair [27], [28], we examined changes in acetylation of histone H3 lysine residues mediated by these two HATs. As p300 HAT is usually involved in global histone H3K27 acetylation and GCN5 HAT functions to regulate global histone H3K9 acetylation [22], [25], human PDAC tumor samples were stained for histone H3K9 and H3K27 acetylation by IHC and trichrome stained to assess for fibrosis. As shown in Figs. 1A and 1B, there is increased histone H3K9 and H3K27 acetylation in regions of fibrosis compared to the non-fibrotic areas. Quantification of the relative staining showed that there was ∼2-fold increase in nuclear staining of histone H3K9 and H3K27 acetylation in areas of fibrosis compared to non-fibrotic areas (Fig. 1C). To determine whether the collagen microenvironment was causally linked to histone H3K9 and H3K27 acetylation, PDAC cells (Panc1 and CD18 cells) were plated on tissue culture plastic or in three-dimensional collagen gels and assessed for histone H3K9 and H3K27 acetylation by Western blotting. PDAC cells grown in three-dimensional collagen gels demonstrated increased histone H3K9 and H3K27 acetylation (Fig. 1D).

We also examined the effect of collagen I-coated surfaces (‘two-dimensional collagen’) on histone H3K9 and H3K27 acetylation. As shown in Supplemental Fig. S1, collagen-coated surfaces had variable effects on histone H3K9 and H3K27 acetylation. Histone H3K9 acetylation was increased on two-dimensional collagen in Panc1 cells, but not in CD18 cells. In contrast, histone H3K27 acetylation was increased on two-dimensional collagen in CD18 cells, but not in Panc1 cells. These results suggest that two-dimensional collagen surfaces can induce, to some extent, histone H3K9 and H3K27 acetylation. However, since the tumor cells in vivo are surrounded by collagen [4], [5], plating cells in three-dimensional collagen is a more representative model to examine the effect of collagen on pancreatic cancer cell behavior.

### HMGA2 regulates collagen-induced H3K9 and H3K27 acetylation

**Figure 2:**
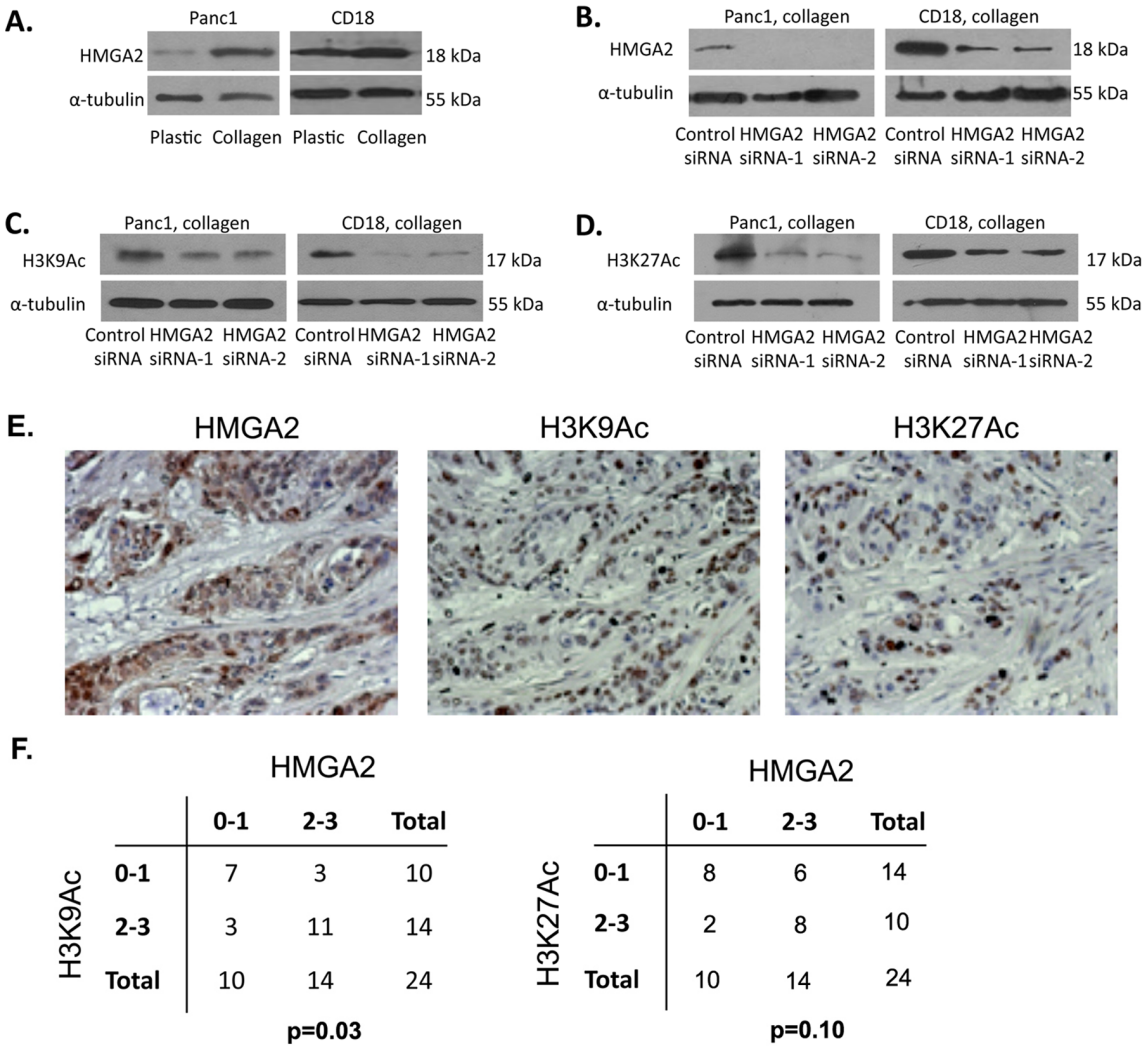
HMGA2 regulates collagen-induced H3K9 and H3K27 acetylation. *A*. Panc1 and CD18 cells were grown on tissue culture plastic or in three-dimensional collagen gels for 24 hours and immunoblotted for HMGA2. The results are representative of three independent experiments. *B*–*D*. Panc1 and CD18 cells were transfected with control siRNA or with 2 different HMGA2 siRNAs, allowed to recover overnight and then embedded in three-dimensional collagen for 24 hours. Lysates were then immunoblotted for HMGA2 (*B*), histone H3K9Ac (*C*) and histone H3K27Ac (*D*) using α-tubulin as loading control. The results are representative of at least three independent experiments. *E*. Human pancreatic TMAs were immunostained for HMGA2 (*left*), histone H3K9Ac (*middle*) and histone H3K27Ac (*right*). *F*. The relationship between HMGA2 and histone H3K9Ac or H3K27Ac was assessed by Fisher's exact test.

We have previously shown that the collagen microenvironment increased HMGA2 expression in PDAC cells [[6] and Fig. 2A]. To determine whether HMGA2 mediated collagen-induced histone H3K9 and H3K27 acetylation, HMGA2 expression was downregulated by 2 different siRNAs in Panc1 and CD18 cells (Fig. 2B) and the effect on histone H3K9 and H3K27 acetylation was determined. As shown in Figs. 2C and 2D, HMGA2 siRNA decreased collagen-induced histone H3K9 and H3K27 acetylation in both Panc1 and CD18 cells. Since our in vitro cultures establish HMGA2 regulation of histone H3K9 and H3K27 acetylation, we next examined the extent to which human PDAC tumor samples that overexpress HMGA2 show evidence of increased histone H3K9 and H3K27 acetylation. As shown in Fig. 2E, human PDAC tumors with HMGA2 expression also demonstrated increased histone H3K9 and H3K27 acetylation. The association between HMGA2 and H3K9 acetylation in our TMAs was statistically significant (p = 0.03), while the association between HMGA2 and H3K27 acetylation trended towards significance (p = 0.10).

### HMGA2 regulates collagen-induced p300, PCAF and GCN5 HAT expression

**Figure 3:**
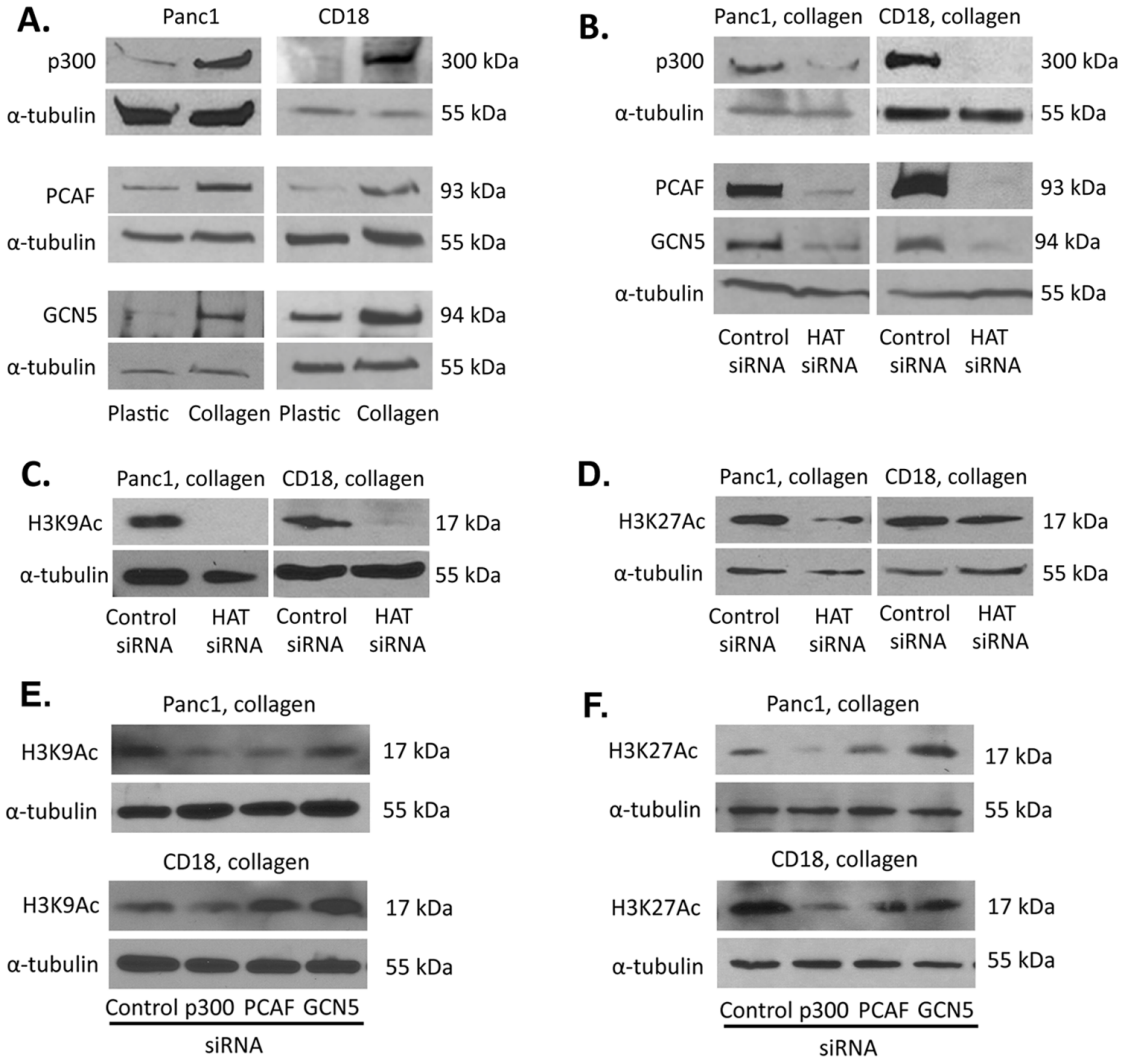
Collagen increases expression of p300, PCAF and GCN5 histone acetyltransferases. *A*. Panc1 and CD18 cells were grown on tissue culture plastic or in three- dimensional collagen gels for 24 hours. Cells were lysed and immunoblotted for p300, PCAF and GCN5 histone acetyltrasferases (HATs) using α-tubulin as loading control. The results are representative of three independent experiments *B*–*D*. Panc1 and CD18 cells were transfected with control siRNA or with combination of siRNAs against p300, PCAF and GCN5 (HAT siRNA); allowed to recover overnight and then embedded in three-dimensional collagen gels for 24 hours. The lysates were immunoblotted for the corresponding HAT proteins (*B*), and histone H3K9Ac (*C*) and H3K27Ac (*D*). The results are representative of at least three independent experiments. *E, F*. Panc1 and CD18 cells were transfected with control siRNA or with individual siRNAs against p300, PCAF and GCN5; allowed to recover overnight and then embedded in three-dimensional collagen gels for 24 hours. The lysates were immunoblotted for histone H3K9Ac (*E*) and H3K27Ac (*F*). The results are representative of three independent experiments.

We next examined the effect of three-dimensional collagen gels on p300, PCAF and GCN5 HATs in Panc1 and CD18 cells. As detailed above, p300 HAT is usually involved in global H3K27 acetylation and GCN5 and PCAF HATs function to regulate global H3K9 acetylation [22], [25]. PDAC cells in three-dimensional collagen gels demonstrate increased expression of p300, PCAF and GCN5 HATs (Fig. 3A). To demonstrate that these HATs in fact mediate collagen-induced H3K9 and H3K27 acetylation in PDAC cells, p300, PCAF and GCN5 expression was downregulated using combination of three different siRNAs in Panc1 and CD18 cells (Fig. 3B), and the effect on H3K9 and H3K27 acetylation was determined. The combination of siRNAs against p300, PCAF and GCN5 decreased collagen-induced histone H3K9 and H3K27 acetylation in both Panc1 and CD18 cells (Figs. 3C and 3D).

We additionally examined the relative contribution of p300, GCN5 and PCAF on histone acetylation using individual siRNAs rather than combining all three siRNAs. As shown in Fig. 3E, transfection of individual siRNAs against p300, PCAF or GCN5 decreased histone H3K9 acetylation in Panc1 cells. However, transfection of the individual HAT siRNAs in CD18 cells had minimal effect or paradoxically increased histone H3K9 acetylation in CD18 cells. Transfection of GCN5 siRNA or PCAF siRNA in CD18 cells increased histone H3K9 acetylation. Interestingly, it was recently shown that there is increased histone H3K9 acetylation in PCAF-null and GCN5-null mouse embryonic fibroblasts [25]. Similarly, the effect on histone H3K27 acetylation was either minimal or increased following transfection of either GCN5 siRNA or PCAF siRNA (Fig. 3F). However, transfection with p300 siRNA reduced histone H3K27 acetylation in both CD18 and Panc1 cells.

**Figure 4:**
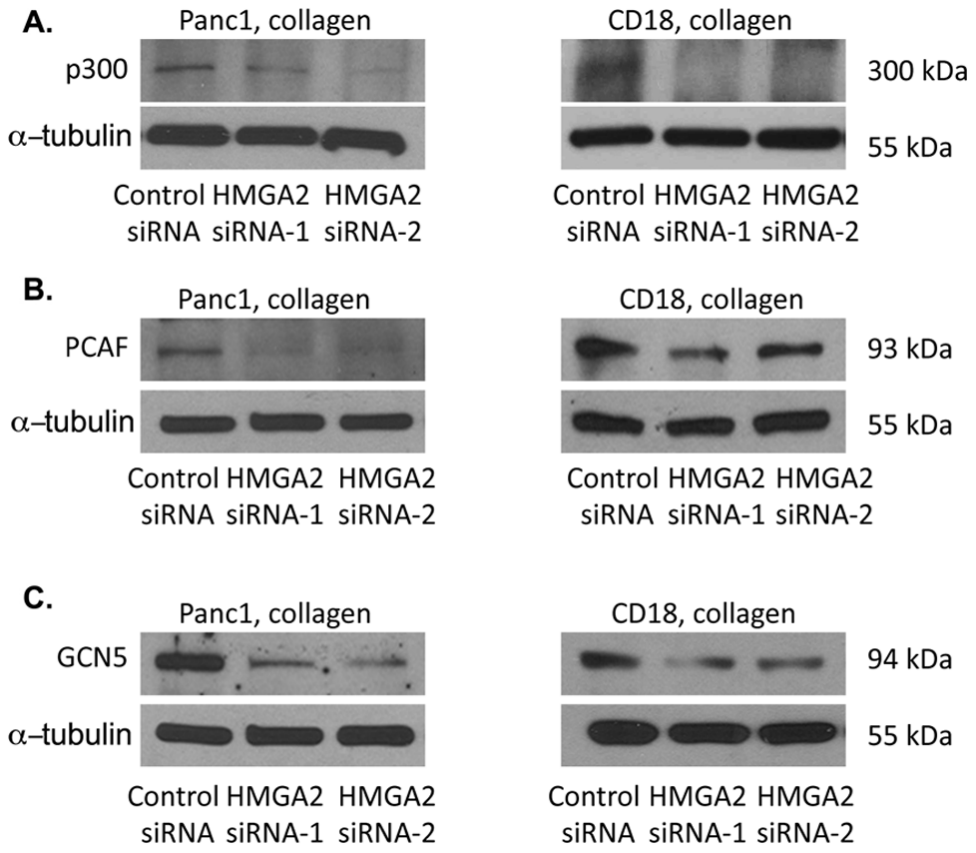
HMGA2 mediates collagen-induced p300, PCAF and GCN5 HAT expression. Panc1 and CD18 cells were transfected with control siRNA or with 2 different HMGA2 siRNAs, allowed to recover overnight and then plated in three-dimensional collagen gels for additional 24 hours. The lysates were then analyzed for p300 (*A*), PCAF (*B*) and GCN5 (*C*) HATs using α-tubulin as loading control. The results are representative of at least three independent experiments.

Since we demonstrate that HMGA2 regulates collagen-induced histone H3K9 and H3K27 acetylation (Fig. 2), we examined the extent to which HMGA2 also mediated collagen-induced p300, PCAF and GCN5 expression. As shown in Fig. 4, HMGA2 siRNA decreased p300, PCAF and GCN5 levels in both Panc1 and CD18 cells grown in 3D collagen.

### PDAC cells in three-dimensional collagen gels are protected against gemcitabine

**Figure 5:**
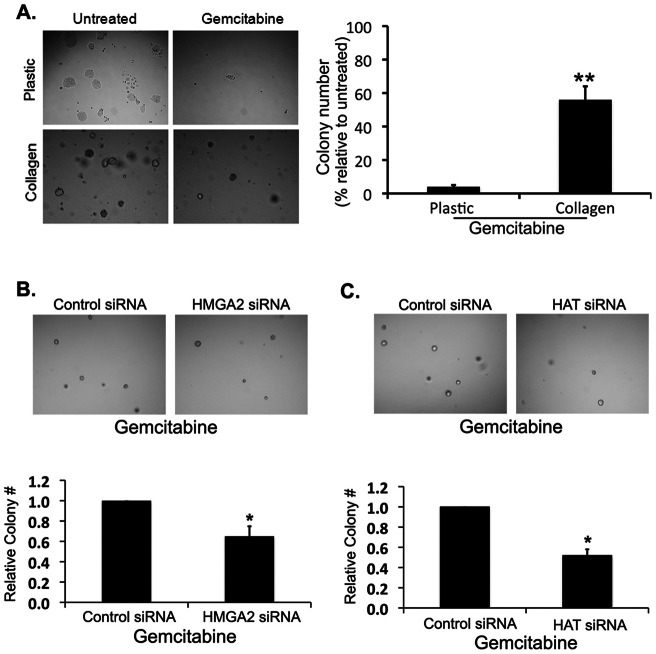
HMGA2 and HATs mediate protection against gemcitabine in three-dimensional collagen gels. *A*. CD18 cells grown on plastic or in three-dimensional collagen gels were left untreated or treated with gemcitabine for 24 hours. The cells were then trypsinized or extracted out of collagen by collagenase treatment. The cells were replated on tissue culture plastic or in three-dimensional collagen gels at low density (*left*). The cells were photographed 5 days later and counted (*right*). **, p<0.001. The results are representative of at least three independent experiments. *B, C*. CD18 cells were transfected with control siRNA, HMGA2 siRNA (*B*) or combination of siRNAs against PCAF, GCN5 and p300 (*C*), allowed to recover overnight and then plated in collagen gels for 24 hours. The cells were then treated with gemcitabine for 24 hours, extracted out of collagen by collagenase treatment and then replated in three-dimensional collagen gels at low density. The cells were photographed 5 days later and counted. *, p<0.05. The results are representative of at least 3 independent experiments.

We had previously shown that PDAC cells in three-dimensional collagen gels were protected against the effects of gemcitabine and continue to proliferate [6]. Thus, we examined the effect of collagen on CD18 cells following gemcitabine treatment. CD18 cells on plastic or in three-dimensional collagen gels were treated with gemcitabine for 24 hours and then trypsinized or subjected to collagenase extraction. The cells were then replated onto plastic or in three-dimensional collagen gels, and the ability of the cells to form colonies was assessed at 5 days. Approximately 5% of CD18 cells on plastic treated with gemcitabine form multi-cellular colonies relative to untreated cells (Fig. 5A). In contrast, greater than 50% of CD18 cells in three-dimensional collagen gels treated with gemcitabine form colonies (Fig. 5A).

### HMGA2 and HATs mediate protection against gemcitabine in three-dimensional collagen gels

As we had previously shown that HMGA2 siRNA decreased proliferation of PDAC cells in three-dimensional collagen gels following gemcitabine treatment [6], we examined the effect of HMGA2 siRNA on PDAC cells in collagen following gemcitabine treatment. CD18 cells were transfected with control siRNA or HMGA2 siRNA, and treated with gemcitabine for 24 hours while growing in three-dimensional collagen gels. The cells were then extracted out of collagen and replated in collagen at a low-density for an additional 5 days. CD18 cells transfected with HMGA2 siRNA show reduced number of colonies compared to CD18 cells transfected with control siRNA (Fig. 5B). Similarly, we examined the effect of siRNAs against p300, PCAF and GCN5 HATs on PDAC cells in three-dimensional collagen gels following gemcitabine treatment. As shown in Fig. 5C, CD18 cells transfected with p300, PCAF and GCN5 HAT siRNAs also show reduced number of colonies compared to CD18 cells transfected with control siRNA.

## Discussion

The collagen-rich tumor microenvironment plays an essential role in PDAC progression by both promoting both tumor invasion and metastasis and protecting cancer cells against chemotherapy [4], [29]. It not only limits the delivery of chemotherapy to cancer cells [7], but it also activates signaling pathways that limit the effect of chemotherapy [6], [7], [8], [9]. Previously, we had published that induction of HMGA2 expression in three-dimensional collagen allowed PDAC cells to overcome the effect of chemotherapy and continue to proliferate [6]. Interestingly, HMGA proteins were recently shown to increase expression of ataxia-telangiectasia mutated (ATM), the main cellular sensor of genotoxic stress, thereby increasing resistance to DNA-damaging agents [30]. In this report, we show that HMGA2 can also regulate the expression of p300, PCAF and GCN5 HATs in three-dimensional collagen gels to limit the effect of gemcitabine.

Although we did not examine whether HMGA2 directly binds to HAT promoters to regulate expression, HMGA2, by acting as a global chromatin switch, has been shown to regulate >1,000 genes [31]. Some of these genes are regulated by HMGA2 by directly binding to the promoter sequences. For example, HMGA2 regulates hTERT expression by binding to the hTERT promoter and causing decreased occupancy of HDAC2 on the hTERT promoter [32]. This leads to a localized increase in histone H3K9 acetylation and transcription modulation of hTERT [32]. However, other studies have shown that HMGA2 can indirectly affect gene expression through activation of signaling pathways, such as the induction of PI3K/Akt/mTOR/p70S6K signaling cascade following overexpression of HMGA2 in stromal cells [33]. We have previously shown that HMGA2 promotes ERK1/2 signaling in pancreatic cancer cells in 3D collagen [6]. Moreover, we have found that ERK1/2 signaling can also mediate collagen-induced HAT expression (Dangi-Garimella S. and Munshi H.G., unpublished observation). Thus, it is possible that HMGA2 regulation of HATs in three-dimensional collagen is mediated through ERK1/2 signaling.

We show that the increased p300, GCN5 and PCAF HAT expression in 3D collagen promotes histone H3K9 and H3K27 acetylation. Importantly, histone H3K9 and H3K27 acetylation are mostly located at transcription start sites and are enriched at promoters of actively transcribed genes [16], [18], and thus changes in histone H3K9 and histone H3K27 can have broad and profound effects on gene expression and cellular behavior. It is possible that the collagen microenvironment may also modulate acetylation of other lysine residues. However, it has been shown that GCN5 and PCAF are redundant and are specifically required for histone H3K9 acetylation in fibroblasts [25]. PCAF-null fibroblasts have preservation of histone H3K9 acetylation and demonstrate reduction in histone H3K9 acetylation only when GCN5 is knocked down in these fibroblasts [25]. Interestingly, the authors convincingly show that GCN5 can acetylate histone H3K14 in an in vitro assay, but no change in histone H3K14 acetylation was detected when PCAF and GCN5 were knocked down in vivo [25]. Moreover, downregulating p300 did not affect histone H3K14 acetylation in vivo, but mainly decreased histone K3K27 acetylation [25]. It is also possible that the changes in histone H3K9 and H3K27 acetylation could be due to repression of histone deacetylases (HDACs) in three-dimensional collagen. HDAC1 and HDAC7 are increased in human pancreatic tumors compared to normal tissue [34], [35]. Moreover, expression of members of class I HDACs in pancreatic cancer cell lines is increased compared to normal HPDE cells [36]. In future studies, we will examine whether the collagen microenvironment modulates expression of HDACs in pancreatic cancer cell lines.

Significantly, we show that the HATs contribute to chemo-resistance in three-dimensional collagen. The p300 HAT has been shown to be involved in the DNA damage response by modulating non-homologous end joining (NHEJ) repair [27]. Decreasing the activity or expression of p300 HAT suppresses NHEJ, impairs double-strand break (DSB) repair and sensitizes lung cancer cells to radiation and chemotherapy [27]. The p300 HAT is required for acetylation of histones at DSBs to facilitate chromatin relaxation and eventual DNA repair [27]. In addition, GCN5 stimulates nuclear excision repair by promoting H3K9 acetylation and chromatin relaxation at sites of damage [28]. Importantly, it is being increasingly recognized that the chromatin state can affect cellular response to chemotherapy. Although the more compact heterochromatin can limit the extent of initial DNA damage [37], it can also restrict access to DNA repair proteins. DNA repair following exposure to carcinogens is less efficient within heterochromatic regions relative to the less compact euchromatic regions [38]. Consistent with the model in which heterochromatin restricts access to proteins involved in DNA repair, treatment with the HDAC inhibitor trichostatin promoted euchromatin formation and increased DNA damage response in breast cancer cells [39].

Although we have not examined the effect of targeting HATs in vivo, our findings strongly suggest that targeting HATs will allow us to increase the efficacy of chemotherapy in mouse models of pancreatic cancer and in patients with pancreatic cancer. This is also supported by the findings that a more open chromatin state in PDAC tumor specimens can be associated with worse prognosis [14], [15]. Although several HDAC inhibitors have been described and extensively studied in cancer progression [40], [41], [42], only a limited number of HAT inhibitors have been developed [22]. Also, it is important to note that PDAC patients treated with the HDAC inhibitor CI-994 and gemcitabine did not demonstrate increased response rates compared to gemcitabine alone [43]. The initial synthetic HAT inhibitors proved effective in blocking HAT activity in vitro; however, their use was limited by low metabolic stability and poor cellular permeability. The use of the naturally occurring HAT inhibitor anacardic acid was also limited by poor cellular permeability [44]. Although the naturally occurring compounds curcumin and garcinol have been shown to be effective HAT inhibitors in vitro and in vivo [22], they also demonstrate significant non-specific activity. Recently, compound C646 was designed by virtual ligand screening and was shown to be a potent, highly selective, cell permeable small molecule p300 HAT inhibitor [45]. The C646 inhibitor impedes intracellular histone acetylation and slows growth of cancer cells in vitro [45], [46]; however, the effectiveness of C646 in animal and human studies has not been reported.

**Figure 6:**
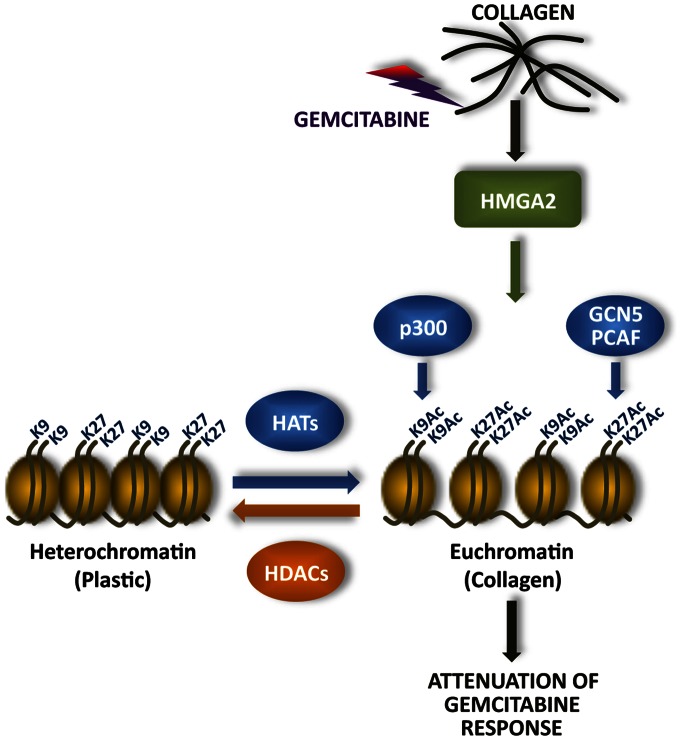
Regulation of gemcitabine resistance by HMGA2 and HATs. Previously, we had published that the collagen microenvironment induced HMGA2 expression [Dangi-Garimella S et al, [6]]. We now demonstrate that PDAC cells in the collagen microenvironment induce HMGA2-dependent HAT expression and histone H3 acetylation, suggesting that the collagen microenvironment promotes euchromatin formation. This signaling pathway allows PDAC cells to resist the effects of gemcitabine in the collagen microenvironment.

Overall, we demonstrate that the collagen microenvironment in vitro limits the effectiveness of gemcitabine through HMGA2-dependent HAT expression (Fig. 6). We also show that HMGA2 expression is associated with histone acetylation in human PDAC tumors, particularly in areas of fibrosis, suggesting that the pronounced fibrotic reaction may contribute to chemotherapy resistance through increased HMGA2-HAT signaling. Given that very little progress has been made in the treatment of pancreatic cancer, targeting HATs could be a novel approach to sensitize pancreatic tumors to chemotherapy.

## Materials and Methods

### Chemicals/Reagents

GCN5 (sc-20698), PCAF (sc-13124), and α-tubulin (sc-8035) antibodies were purchased from Santa Cruz Biotechnology (Santa Cruz, CA); p300 (05–257), histone H3K9 acetylation (04–1003), and histone H3K27 acetylation (05–1334) antibodies were obtained from Millipore (Billerica, MA); and HMGA2 antibody was from Biocheck Inc. (Foster City, CA). Secondary antibodies were purchased from Sigma (St. Louis, MO). Type I collagen was obtained from BD Biosciences (Franklin Lakes, NJ). Gemcitabine was obtained from Eli Lilly (Indianapolis, IN). Nucleofector electroporation kit was purchased from Lonza (Walkersville, MD). HMGA2 #1 (279254), HMGA2 #2 (279255), GCN5 (s5659), PCAF (s16894), and p300 (s4696) siRNAs were purchased from Life Technologies (Carlsbad, CA).

### Immunohistochemistry (IHC)

Pancreatic tissue microarrays (TMAs) were obtained from U.S. Biomax (Rockville, MD) and consisted of 24 pancreatic cores measuring 1.5 mm in diameter and 5 μm in thickness. The slides were trichrome stained or stained for H3K9 acetylation, H3K27 acetylation, and HMGA2 according to standard IHC procedures [5], [6], [47]. Stained samples were graded on a scale of 0–3 with the following scoring system: 0–0% of the cells stained; 1–<25%; 2–26–50%, or 3–>50%.

### Cell culture

Panc1 and CD18/HPAF-II were obtained from ATCC (Manassas, VA). Cells were maintained in DMEM containing 10% FBS and antibiotics (100 U/ml Penicillin and 100 µg/ml Streptomycin) [6]. The cells were tested by STR profiling at the Johns Hopkins Genetic Resources Core Facility and showed a similar profile to that on the ATCC website.

### Embedding cells in three-dimensional type I collagen gels

Collagen mixture (2 mg/mL) was made by adding the appropriate volumes of sterile water, 10X DMEM and NaOH and kept on ice until needed [6], [48], [49]. PDAC cells were suspended in the collagen solution and allowed to gel for 15 minutes at 37°C. Regular media was then added on top of the gel and incubated for 24 hours.

### Transfection

Cells were transfected with siRNA against HMGA2, GCN5, PCAF, p300 or control siRNA (50 nmoles) using Nucleofector Kit R (Lonza), allowed to recover overnight and then plated in three-dimensional collagen gels (2 mg/ml).

### Immunoblotting

Immunoblotting was done as previously described [5], [50]. For cells grown in collagen, the matrix was first dissolved in collagenase (Worthington Biologicals, Lakewood, NJ) and then lysed as previously described [6], [51].

### Colony forming assay

CD18 cells on tissue culture plastic or in three-dimensional collagen gels were treated with gemcitabine. Twenty-four hours later, the cells were trypsinized or extracted out of collagen, and then replated on tissue culture plastic or in three-dimensional collagen gels at a low density. The cells were photographed 5 days later and counted.

### Statistical analysis

Statistical analyses were done with GraphPad InStat (LaJolla, CA), using t-test analysis or Fisher's exact test.

## Supporting Information

Figure S1 **Effect of 2D collagen on histone H3K9 and histone H3K27 acetylation.** *A, B*. Panc1 and CD18 cells were grown on tissue culture plastic or on collagen I-coated tissue culture plates (BD BIocoat Collagen I) for 24 hours. Cells were lysed and immunoblotted for histone H3K9Ac and H3K27Ac using α-tubulin as loading control. The results are representative of three independent experiments.
(TIF)
