# Supplementary figures and images for: Three-Dimensional Collagen I Promotes Gemcitabine Resistance In Vitro in Pancreatic Cancer Cells through HMGA2-Dependent Histone Acetyltransferase Expression
Source: PLoS One. 2013 May 16;8(5):e64566. doi: 10.1371/journal.pone.0064566 (PMC3655998; doi:10.1371/journal.pone.0064566)

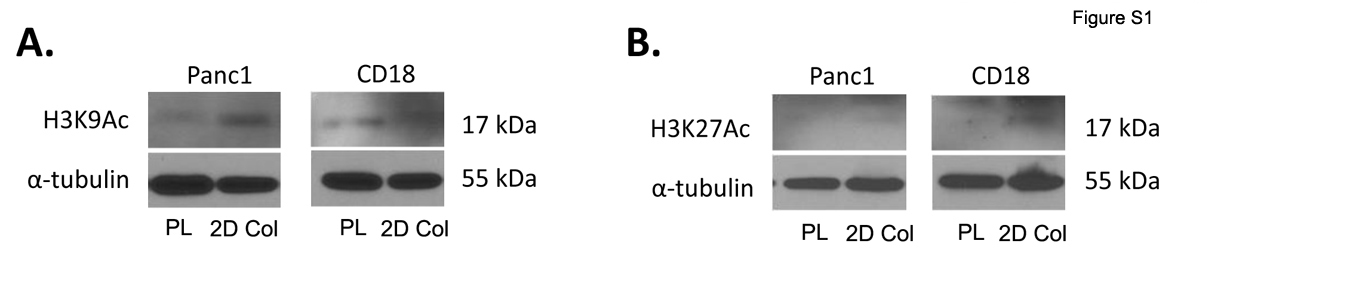

Supplement: Figure S1 — Effect of 2D collagen on histone H3K9 and histone H3K27 acetylation. A, B. Panc1 and CD18 cells were grown on tissue culture plastic or on collagen I-coated tissue culture plates (BD BIocoat Collagen I) for 24 hours. Cells were lysed and immunoblotted for histone H3K9Ac and H3K27Ac using α-tubulin as loading control. The results are representative of three independent experiments. (TIF) [file pone.0064566.s001.tif]
